# Primary Hepatic Neuroblastoma in a 5.5-Month-Old Boy: A Case Report

**DOI:** 10.34172/aim.2024.07

**Published:** 2024-01-01

**Authors:** Farzad Kompani, Alieh Safari Sharari, Elmira Haji Esmaeil Memar, Mahya Ghahremanloo

**Affiliations:** ^1^Division of Hematology and Oncology, Children’s Medical Center, Pediatrics Center of Excellence, Tehran University of Medical Sciences, Tehran, Iran; ^2^Department of Pediatrics, Pediatrics Center of Excellence, Children’s Medical Center, Tehran University of Medical Sciences, Tehran, Iran

**Keywords:** Liver neoplasms, Neuroblastoma, Pediatrics

## Abstract

The most frequent type of extracranial solid tumor in pediatric cases is neuroblastoma (NB), almost always arising in tissues with sympathetic innervation with only a few reported cases arising in other organs. NBs with hepatic involvement are typically metastatic lesions as primary hepatic NBs are extremely rare. This study presents a 5.5-month-old boy with primary hepatic NB. This case study describes a male 5.5-month-old preterm infant who presented with overt hepatomegaly. Laboratory tests showed an abnormally high level of alpha-fetoprotein. A sonography-guided liver needle biopsy was performed, so histopathological examination suggested the diagnosis of a small round-cell tumor. Immunohistochemical staining demonstrated evidence of neuronal differentiation in the tumor. The sum of these findings was in favor of the diagnosis of NB. Bone marrow aspiration and biopsy were normal. The full-body computed tomography scan revealed a large intrahepatic mass measuring 82×70×74 mm with mild peripheral enhancement. A metaiodobenzylguanidine (MIBG) scintiscan confirmed a huge round MIBG-avid hepatic lesion without other remarkable lesions at other sites in the body. Chemotherapy treatment was started for the patient, and after 4 sessions of chemotherapy, an ultrasound showed that the mass size had decreased to 55×36 mm. This report describes the first primary hepatic NB in a pediatric patient with detailed clinicopathological details. Primary hepatic NB is extremely rare. It is important to consider neuroendocrine tumors as a possibility when faced with a single hepatic tumor that has a similar histological appearance.

## Introduction

 The most frequent type of extracranial solid tumor in pediatric cases is neuroblastoma (NB).^1^ This type of tumor is a neuroendocrine tumor that originates from progenitor cells found in the neural crest during embryonic development,^2^ and almost always arises in tissues with sympathetic innervation, their most common locations being in the superior cervical, paraspinal, and celiac ganglia, and the adrenal glands ^3^. NB originating from organs outside of the sympathetic nervous system has only been documented in a small number of cases.^4^ The annual incidence of NB is 65 patients per million infants in the United States.^3^ Hepatic NBs are considered metastatic lesions and are included as stage 4S of the International NB Staging System (INSS) of adrenal gland NBs.^5^ Primary hepatic NB is extremely rare, with the only other case report being a 29-year-old woman with a primary hepatic NB.^6^ This study aims to present a 5.5-month-old boy with a primary hepatic NB.

## Case Report

 A 5.5-month-old male preterm infant with hepatomegaly presented to the Pediatrics Center of Excellence Hospital. The child was born by cesarean at 35 weeks and 6 days of gestation, and was the third child in a family of five. His two older siblings were normal, there was no history of abortion in the mother, and familial history and drug history were otherwise unremarkable. Clinical examination revealed the isolated finding of massive hepatomegaly. In fetal assessments, evidence of bilateral hydronephrosis was reported on ultrasound, and further evaluation was recommended at postnatal follow-up visits.

 Initial laboratory tests, including complete blood counts, a biochemistry panel, and liver and kidney function tests, demonstrated no remarkable findings. Abnormally high levels of alpha-fetoprotein equal to 133 ng/mL were found in further laboratory studies. A large mass in the liver was described on abdominal and pelvic ultrasound. Brain sonography and chest X-ray were normal.

**Figure 1:**
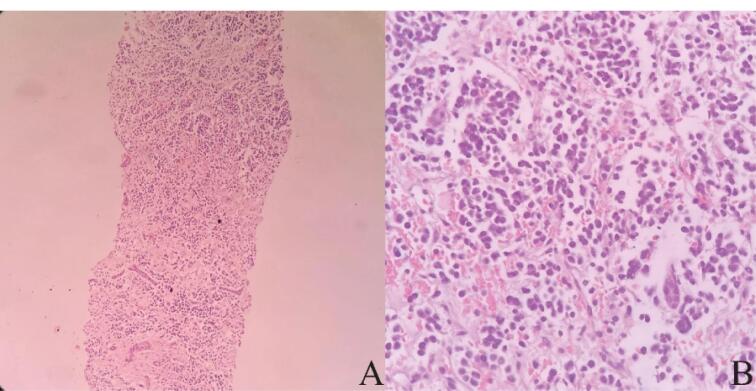
Pathologic Specimens. *Note*. Microscopically, the tumor consists of nests of small round cells with intervening fibrovascular bundles. These cells have round nuclei, coarse chromatin, scant cytoplasm, high N/C ratio, and inconspicuous nuclei. Mitotic figures are detected. A: H&E, × 40, B: H&E, × 100

 A sonography-guided liver needle biopsy was performed. Microscopically, the sections showed neoplastic tissue composed of nests of small round cells with intervening fibrovascular bundles. These cells had round nuclei, coarse chromatin, scant cytoplasm, high N/C ratio, and inconspicuous nuclei. Mitotic figures were found as well (Figure 1). Based on these findings, the diagnosis of a small round cell tumor was made for the patient. Immunohistochemical staining was performed to type the nature of the neoplasm. The tumor cells demonstrated a significant level of reactivity to synaptophysin and CD56, supporting the notion of neuronal differentiation. No cytokeratin, desmin, and HepPar1 expressions were detected. Immunohistochemical results and tumor morphology supported the diagnosis of NB.

**Figure 2:**
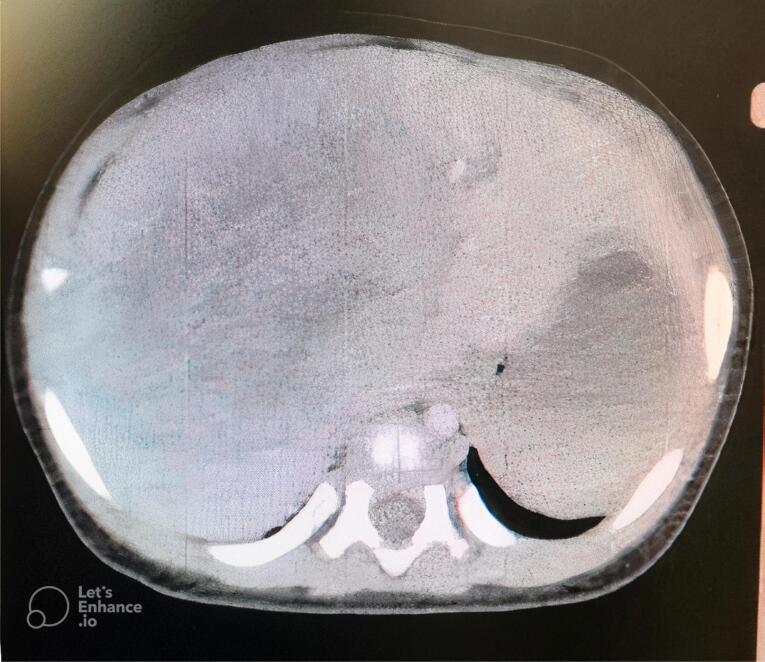
An Abdominal Computed Tomography Scan Image. *Note*. There is a large intra hepatic mass measured 82 × 70 × 74 mm with mild peripheral enhancement

 Considering that primary hepatic NB is highly rare, a systemic examination was performed to identify possible primary tumor deposits. Bone marrow aspiration and biopsy were normal. Whole-body computed tomography scan revealed a large (82 × 70 × 74 mm) intrahepatic mass which displaced hepatic vessels, the biliary system, and the right kidney (Figure 2). The mass showed mild peripheral enhancement after intravenous injection. A metaiodobenzylguanidine (MIBG) scintiscan was performed, and there was a large, round, MIBG-avid hepatic lesion with central photopenia, without other remarkable lesions in other sites of the body.

 After confirmation of the diagnosis of primary hepatic NB, the patient was started on a chemotherapy regimen. After 4 sessions of chemotherapy with an interval of 21 days (using vincristine, doxorubicin, and cyclophosphamide #3/cisplatin and etoposide #1), an ultrasound was performed for the patient, representing that the mass size had decreased to 55 × 36 mm.

## Discussion

 We reported a 5.5-month-old boy with primary hepatic NB. This case was unique because of the rarity of primary hepatic NB. As the available reported case, this is only the second documented case of primary hepatic NB and the first involving a child. Primary hepatic NB is a very rare presentation of NB that was reported only in one patient; Jung and Kim reported a 29-year-old woman with primary hepatic NB.^6^

 The most frequently occurring extracranial solid tumor in children is NB, responsible for 7.8% of all pediatric cancers. At the time of diagnosis, over 50% of patients have already advanced metastatic tumors.^5,7^ Metastatic hepatic NB is a rare presentation of NB, and it is known as stage 4 in the INSS of NB.^8^ NB stage 4s accounts for about 7%–10% of all NB cases,^9^ implying that metastatic hepatic NB is a rare disorder in the field of pediatrics.

 NB most frequently affects the adrenal gland, followed by the neck, mediastinum, and pelvis. On the other hand, NB that originates in the liver is usually detected only after a primary adrenal tumor has already metastasized to the liver. There has been one other recorded instance of solitary hepatic NB, though it was in fact a metastatic lesion, due to delayed metastasis after treatment and full remission of a primary adrenal NB.^10^

 The origins of primary hepatic NBs are still uncertain. However, it is known that the sympathetic nervous system plays a significant role in glucose metabolism and tissue repair in the liver due to its innervation of the organ; NB might develop from intrahepatic sympathetic nerve cells.^11,12^

 When patients are diagnosed with NB, 40% are under the age of one year. Around 35% of cases occur in children between the ages of one and two, while 25% are found in those over two years old.^13^ Our patient was 5.5 months old, which falls into the most common age category of age less than 1 year old.

 Although some tumors are discovered in asymptomatic newborns during normal evaluation, it is most commonly diagnosed after the detection of a palpable abdominal mass or abdominal distention in an infant.^14,15^ In our patient, hepatic NB was diagnosed incidentally, and the patient was asymptomatic, with the only remarkable finding being hepatomegaly in the physical examination.

 The presence of NB metastasis may be determined with great precision and accuracy using sympathetic neuronal absorption of MIBG, which is comparable to norepinephrine.^16^ In the course of the evaluation of this patient, bone marrow biopsies were taken, as they are necessary for tumor staging and determination of bone marrow involvement.^2^ An MIBG scan was then performed to evaluate other potential sites of NB. We suspected the presence of another focus of NB and performed these studies in search of a primary site and in the interest of staging the disease. However, no other sites of NB were detected, neither on bone marrow biopsy nor in the MIBG scan.

 The prognosis for NB in the high-risk category (primary tumor with spread to liver, skin, or bone marrow) is the worst (except for type 4s), with extensive metastatic disease affecting the bone marrow, bone, lungs, and liver. Patients will undergo induction chemotherapy to minimize tumor size at both primary and metastatic sites, followed by maximum surgical resection, myeloablative chemotherapy, and stem-cell transplantation. Patients are then treated with a mix of maintenance chemotherapy and immunotherapy.^17^ The treatment of high-risk NB has become much more successful with intensive and multi-faceted approaches, including chemotherapy, surgery, radiation therapy, myeloablative chemotherapy followed by stem cell rescue, and immunotherapy.^18^ The International NB Risk Group Staging System is widely used by major international groups and the Children’s Oncology Group as a means of assessing risk.^19^ Precise risk assessment is crucial for determining the most effective surgical treatment. Neonatal adrenal NBs and metastatic tumors may be monitored in some cases, while very low-risk and low-risk NBs can be treated with surgery exclusively. However, intermediate-risk tumors often necessitate systemic chemotherapy.^20^

 Our patient underwent chemotherapy, and the size of the hepatic mass decreased. The patient is still receiving chemotherapy and is in good general condition.

## Conclusion

 In this report, the first primary hepatic NB in a pediatric patient was described with detailed clinicopathological details. Although primary hepatic NB is an uncommon occurrence, it is important to consider it as a possible diagnosis when presented with a singular tumor in the liver that bears similar histological characteristics to a neuroendocrine tumor.
